# Notes and News

**Published:** 1891-04-11

**Authors:** 


### Notes and News.

Collected and Communicated.

On the 3rd inst., H.R.H. the Duke of Connaught was present at a meeting of the Committee of the Royal Portsmouth, Portsea, and Gosport Hospital, and afterwards went over the wards, being accompanied by the Chairman (Sir William King), Dr. J. Ward Cousins, Dr. Lloyd-Owen, and Dr. Rundle, members of the honorary medical staff.

At a public meeting in Fleetwood, it was decided to establish a Cottage Hospital in the town, a house for this purpose having been presented by Mr. C. H. Smith. The cost of furnishing and fitting up the house, which will contain four beds for men and two for women, is estimated at £200. The Hospital Aid Society are of opinion that it can be maintained for £200 per annum, towards which there is a visible income of £160, leaving £40 to be made up by outside subscriptions each year.

The Board of Management of the Manchester Infirmary have decided to decline the offer made by the authorities of Owens College, of a site in Stanly Grove, for a Branch Hospital, which was to be built instead of a new wing to the Infirmary. The Infirmary Board have already to administer four institutions, and they naturally shrink from establishing a fifth. They have motives of convenience and economy for an extension on their present site, and they decline to appropriate any existing funds, or to appeal for fresh support, in order to establish a branch elsewhere.

Meanwood Convalescent Home for Children, which was re-opened on March 1st, 1890, has admitted since then 222 little patients. The general health of the children has been very satisfactory, and the average gain in weight of each child was over three pounds. The cost per head for the term of three weeks was £1 10s. 4d.; the total expenditure for the year being £436 19s. 3d., as against receipts amounting to £474 1s. 1d. During the last two years the home has lost 20 subscribers through death or removal from Leeds. Additional subscribers and also presents of old clothes and boots and shoes are urgently needed.

Although it is only twelve years since the opening of the new Royal Infirmary buildings in Edinburgh, this splendid edifice is already declared to be inadequate to meet the necessities of the ever-increasing population. The number of patients admitted has increased enormously during the last ten years, and in nine months out of the twelve the Infirmary has constantly to refuse admission to large numbers of persons applying for treatment. The amount of clinical teaching, also, which can be given is hardly sufficient for the number of students, and in consequence there is often a class of students twice larger than the number of patients treated. The managers have had the question of extension under consideration for some time, and meanwhile have obtained a little additional space within the Infirmary by erecting a new home for nurses. Extension can only be made westward, which involves the purchasing of the entire property in Lauriston Lane.

The Empress Frederick paid a private visit on Saturday last to the Throat and Ear Hospital, in Golden Square. Her Majesty was received on her arrival by Sir Morell Mackenzie, Lord Calthorpe, the President, and Mr. C. Welch, the Chairman of the Committee, and Mr. F. Horniman, who so liberally contributed towards the establishment of the new children's ward now about to be opened. The Empress, who naturally takes a great interest in the institution, visited all the wards, and spoke to each of the patients, expressing her satisfaction at the general cheerfulness with which they were surrounded. Before leaving, the Empress was presented with a bouquet by the youngest patient, a boy on whom the operation for tracheotomy had been performed.

Mr. Dale's scheme for a new hospital for Scarborough has been adopted at the general meeting held for its consideration. The present hospital was originally established as a dispensary only, and is not now sufficient for the necessities of the town. There is no separate ward for children, no proper provision for paying patients, and no accommodation for extra nurses when they are required. There is no separate room for operations, nor proper accommodation in the way of baths or lavatories. The consulting room is far from what it should be. There are, therefore, good and substantial reasons for urgency in obtaining a new and better equipped building. A suitable site has been found, on which there are buildings to the value of £2,000, which can be adapted to form part of the hospital. The cost of the site, alterations, new buildings, &c., is estimated at £6,225, against which there is the value of the present hospital. The endowment fund now amounts to about £6,250, but the building will not be begun until it amounts to £10,000.

The one hundred and eleventh annual meeting of the Governors of the Birmingham General Hospital was held on March 18th the Hon. A. G. C. Calthorpe in the chair. The report submitted by the Committee showed that the total number of patients who received relief during 1890 was greater than during any year of the hospital's work. This increase in the number of patients, and the absence of special sources of income from the Musical Festival and the Hospital Sunday collection, has resulted in an adverse balance of £6,073 1s. 8d. in the funds of the hospital. Considering the additional work of the hospital the economy of the expenditure showed a marked improvement in most departments, especially in that of housekeeping, there being a decrease of £458 8s. 7d. under this head alone. In moving the adoption of the report the Chairman, alluding to the falling-off shown in the subscription list, said in a town like Birmingham it should show continual increase. In seconding the motion, Mr. Holder announced that the second list of donations to the building fund would show that over £84,000 had been promised. The report was adopted.

One of the worst parts of the volunteer service is the medical staff. If there had really been a battle at Dover this Easter, we should have been sorry for the wounded. It is all very well to say that England is supplied with hospitals to which the wounded could be taken, but these hospitals hold the diseased, and a sudden rush of, say, one hundred wounded to any hospital in the Kingdom could not be properly met. There were some half-dozen casualties amongst the London Scottish during the manœuvres—one man got a bit of glass in his eye, another cut his knees badly, and so on, and there was no surgeon with the regiment. It has been pointed out before by abler pens that it is a pity not to perfect the medical branch of the volunteer force, and something has been done, but not enough. Present arrangements might meet the difficulty of a few minor accidents, but could in no way cope with five or six hundred wounded from a battle-field.

